# Functional efficiency of PCR vectors *in vitro* and at the organism level

**DOI:** 10.1371/journal.pone.0232045

**Published:** 2020-04-24

**Authors:** Dina R. Safina, Polina I. Selina, Marina P. Roschina, Maria A. Karaseva, Alexey A. Komissarov, Ilya V. Demidyuk, Eugene D. Sverdlov, Sergey V. Kostrov

**Affiliations:** Department of Molecular Genetic Fundamentals of Biotechnology and Protein Engineering, Institute of Molecular Genetics, Russian Academy of Sciences, Moscow, Russia; Imperial College London, UNITED KINGDOM

## Abstract

The functional efficiency of the expression cassettes integrated into a plasmid and a PCR- amplified fragment was comparatively analyzed after transient transfection *in vitro* or introduction into the developing embryo of *Danio rerio*. The cassettes contained the reporter genes, luciferase of *Photinus pyralis* (*luc*) or enhanced green fluorescent protein, under the control of the promoter of human cytomegalovirus immediate-early genes. In the *in vitro* system, the efficiency of the circular plasmid was 2.5 times higher than that of the PCR- amplified fragment. The effect of mutations in the expression cassette on the efficiency of the transgene expression in the PCR- amplified fragment was quantitatively evaluated. The mutations generated after 25 amplification cycles with *Taq* DNA polymerase decreased luciferase activity in transfected cells by 65–85%. Thus, mutations are the key factor of decreased functional efficiency of the PCR- amplified fragment relative to the circular plasmid in this experimental model, while other factors apparently have a lesser impact. At the organism level, no significant difference in the expression efficiency of the plasmid and PCR- amplified fragment has been revealed. Comparison of the vector efficiencies in *in vivo* and *in vitro* systems demonstrates that the level of luciferase in the *D*. *rerio* cell lysate, normalized to the molar concentration of the vector, is by three orders of magnitude higher than that after the cell transfection *in vitro*, which indicates that the quantitative data obtained for *in vitro* systems should not be directly extrapolated to the organism level.

## Introduction

Considering the safety concern of gene therapy in clinical practice, the development of minimal genetic vectors is among the key approaches to optimize the expression constructs [1–7].

This strategy is primarily intended to exclude functional elements such as antibiotic resistance genes, bacterial replication origin sites, nonspecific transcription initiation sites, unmethylated CpG dinucleotides, etc. [8–15]. In this context, vectors based on PCR- amplified DNA fragments are of considerable interest [16–18]. This approach makes it possible to strictly control the size and structure of the genetic construct and to limit its size to that of the minimal expression cassette when required.

The genetic constructs based on PCR fragments can provide for active and long-term expression of transgenes in various model systems; [1, 19–25] however, many aspects of PCR- amplified fragment functioning remain underexplored.

For instance, the quantitative contribution of the size, topology, and sensitivity to endonucleases of the amplified DNA fragment to the transgene expression relative to plasmid constructs remain unclear.

The quantitative impact of mutations in the expression cassette acquired during PCR on the expression efficiency of the vector is also obscure. Addressing these problems is critical to optimize the expression vectors.

In this context, it should be stressed that using *in vitro* cell cultures and organism level systems can provide for substantially different results [15, 24]. This is due to the fact that *in vitro* and *in vivo* systems are different fundamentally in the nature of factors that influence the functioning of expression genetic constructs.

In a simpler *in vitro* system, the key factors of the vector efficiency are the capacity to penetrate cells, to release from endosomes, to transport into the nucleus, and to realize the expression of target genes. Largely, these factors determine the structural and functional elements of the vectors required for their efficient application.

At the same time, the transgene expression in an *in vivo* system is substantially affected by other factors such as the method of their introduction, stability in the bloodstream, rate and mechanisms of removal from the body, etc., which are absent in the case of direct transfection of cultured cells.

It should be noted that the introduced vectors interact with a variety of cell populations. Both the penetration of vectors into these cells and their functioning can be carried out according to different scenarios.

It is obvious that the contribution of all these factors, which must be taken into account to optimize the structural organization of the expression constructs as well as the modes of their application, cannot be estimated using the in vitro system. At the same time, *in vitro* systems emphasize the significance of processes directly linked to the intracellular transport of the constructs and the efficiency of the expression cassettes.

Here, we comparatively analyzed the expression efficiency of plasmid genetic vectors and PCR- amplified fragment ones after transient transfection of cells or introduction into the developing embryo of *D*. *rerio*. The *P*. *pyralis* luciferase and the green fluorescence protein genes were used as the marker transgenes.

## Materials and methods

### Plasmid constructs

**Fig 1:**
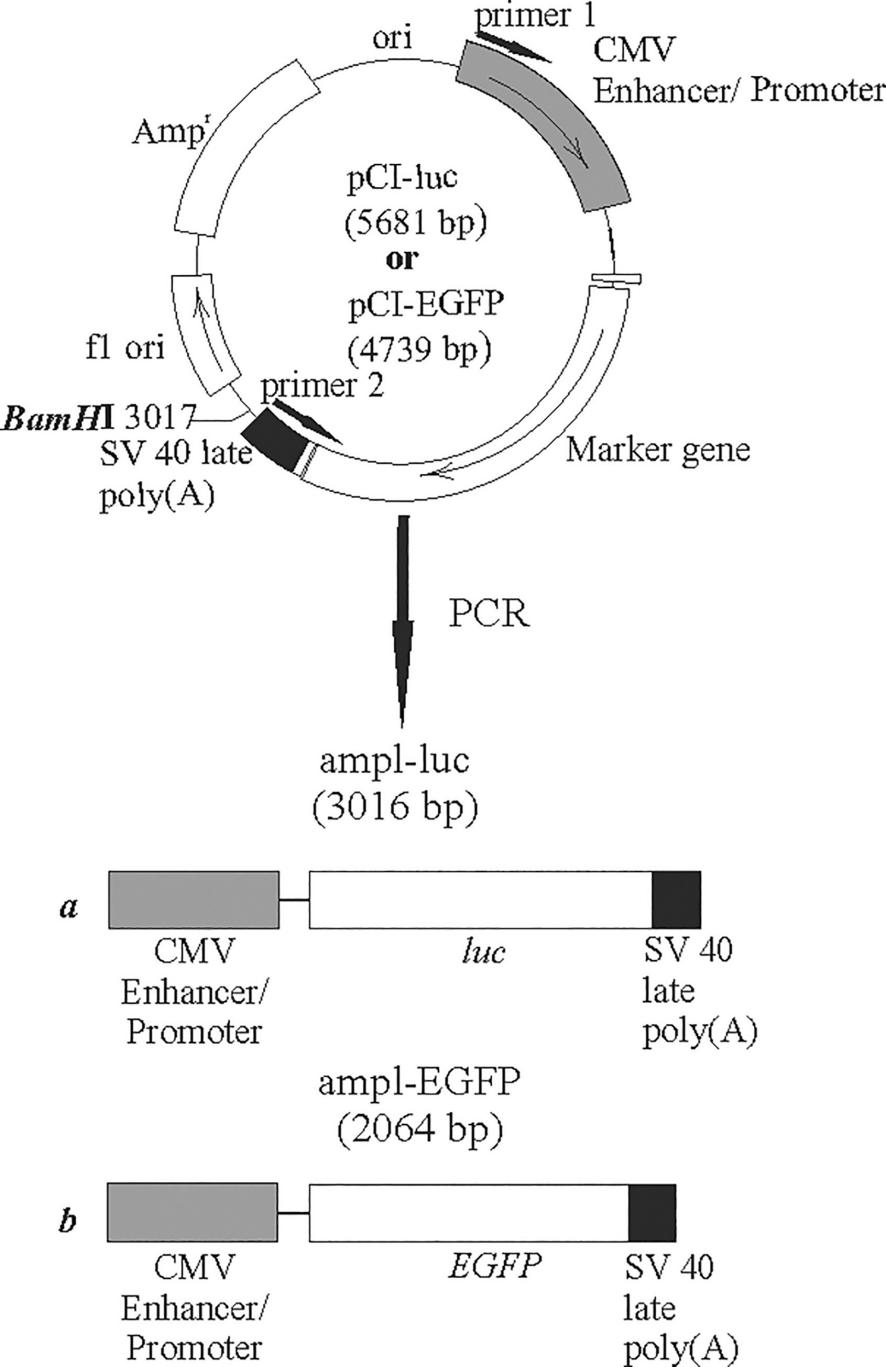
The structures of the genetic constructs used. pCI-luc and pCI-EGFP plasmids contain the luciferase gene (*luc*) of firefly *P*. *pyralis* and green fluorescent protein (*EGFP*), respectively. CMV Enhancer/Promoter, enhancer and promoter of the human cytomegalovirus immediate-early genes; SV40late poly(A), polyadenylation signal of SV40 late genes.

The plasmid pCI-EGFP (Fig 1) was constructed previously by cloning the *EGFP* gene-containing fragment from pEGFP-N1 (Clontech, Mountain View, CA, USA) into the *Kpn*I and *Not*I sites of pCI (Promega, Madison, WI, USA) [24]. The plasmid size was 4739 bp.

The pCI-luc plasmid was constructed by cloning the *P*. *pirales* luciferase gene from pGL3-control (Promega, Madison, WI, USA) into the *Hind*III/*Eco*RI-*Xba*I sites of pCI (Promega, Madison, WI, USA). The pGL3-control vector was digested with *Hind*III, filled in with Klenow polymerase, and treated with *Xba*I.

The pCI plasmid was digested with *Eco*RI, filled in with Klenow polymerase and treated with *Xba*I. The resulting construct size was 5681 bp.

pCI-luc**_**lin was linearized at the *Bam*HI site located 11 bp downstream of the polyadenylation site. After electrophoresis in 1% agarose gel containing 89 mM Tris-HCl, 89 mM boric acid, and 2 mM EDTA, pH 7.6, the linearized plasmid was isolated from the gel using the Cleanup Standard Kit (Eurogene, Moscow, Russia).

The enzymes and buffer solutions were purchased from SibEnzyme (Moscow, Russia).

DNA was quantified using the extinction coefficient of 0.02 ml/(μg×cm) for double-stranded DNA [26].

### PCR- amplified constructs

The genetic constructs ampl-luc and ampl-EGFP (Fig 1) were generated by PCR and containing the expression cassettes similar to those described above. The pCI-luc plasmid was used as a template for the ampl-luc construct. The PCR primers were designed to provide the amplification of the pCI-luc fragment from the beginning of the promoter to the end of the polyadenylation site (Fig 1). The amplification product size was 3016 bp.

The reaction mixture (50 μl) contained 0.03 pmol of pCI-luc as the template, 20 pmol of the primers TCAATATTGGCCATTAGCC and ACCACATTTGTAGAGGTTTTAC (Eurogene, Moscow, Russia), 15 nmol dNTPs each, 2 U of *Taq* DNA polymerase, and 5 μl of the reaction buffer (SibEnzyme, Moscow, Russia). Amplification was conducted on a Tercik amplifier (DNA technology, Moscow, Russia) using the following program: 94°C for 4 min; 10 to 35 cycles of 94°C for 30 s, 55°C for 30 s, 72°C for 3 min; and 4°C hold.

Ampl-EGFP was generated using pCI-EGFP as the template and the amplification program of 94°C for 4 min; 25 cycles of 94°C for 30 s, 55°C for 30 s, 72°C for 2 min 10 s; and 4°C hold. The size of the amplification product was 2064 bp.

In all cases, the PCR products were fractionated, and their concentration was determined as described for the linear fragment of pCI-luc.

Constructs p1–p10 were generated by cloning the Klenow’s fragment treated products of ampl-luc amplification into the *Hind*II site of pUC19. Enzymes and buffers purchased from SibEnzyme (Moscow, Russia) were used.

The cloned DNA fragments of the p1, p2, p6, and p7 plasmids were sequenced by the Genom Service (Engelhardt Institute of Molecular Biology, Russia) using the primers GTAAAACGACGGCCAGT, GCCTTTCTCTCCACAGG, and CAGGAAACAGCTATGAC (Eurogene, Moscow, Russia).

### Transformation of *E*. *coli*

The plasmid constructs were introduced into *E*. *coli* Tg1 cells by electroporation using a MicroPulser electroporator (Bio-Rad Laboratories, Hercules, CA, USA).

#### Plasmid DNA isolation

Was performed using the Plasmid Miniprep Kit (Eurogene, Moscow, Russia).

### Human cell culture and transfection

Human embryonic cells HEK293 (from the Russian Collection of Cell Cultures, St. Petersburg, Russia) was cultured in DMEM/F12 (1:1 v:v) (Paneco, Moscow, Russia) supplemented with 10% embryonic fetal bovine serum (FBS) (GE Healthcare, Chicago, Illinois USA) and 0.3 mg/ml glutamine (MP Biomedicals, Irvine, CA USA) at 37°C under 5% CO_2_. Twenty-four hours prior to transfection, the cells were transferred to a 48-well plate (SPL Life Sciences, South Korea) at 50,000 cells per well. The cells were quantified using a Countless II FL counter (Life Technologies, Carlsbad, CA USA). The cells were transfected at 70% confluence.

The transfection mixture was prepared according to the manufacturer’s recommendations (Thermo Fisher Scientific, Waltham, Massachusetts USA), 50 μl of serum-free OptiMEM (Thermo Fisher Scientific, Waltham, Massachusetts USA) were supplemented with 500 ng of total DNA and 1 μl of the TurboFect transfection agent (Thermo Fisher Scientific, Waltham, Massachusetts USA).

The cells with the transfection mixture were incubated for 24 h, the medium was replaced with DMEM/F12 containing 10% of FBS and 0.3 mg/ml of glutamine, and the cells were incubated for another 24 h, after which total protein and luciferase activity were assayed in cell lysates.

At least five independent experiments with at least three replicates were carried out for each analyzed genetic constructs and DNA transfection doses.

### Preparation of HEK293 and *D*. *rerio* cell lysates

HEK293 cells were lysed following the recommendations for the Luciferase Assay System (Promega, Madison, WI, USA).

*D*. *rerio* embryos were dechorionated and anesthetized with 0.006% water solution of tricaine (Sigma-Aldrich, St. Louis, MO, USA). Then, 3–5 embryos were placed in a microcentrifuge tube (cat.# 1260–00, Scientific Specialties, Lodi, CA USA) removing as much of tricaine solution as possible, and 50 μl of the lysis buffer (Luciferase Assay System Kit, Promega, Madison, WI, USA) were added together with glass powder (Serva Electrophoresis, Heidelberg Germany). The embryos were homogenized using an Eppendorf micro-pestle (cat.# 0030120973, Thermo Fisher Scientific, Waltham, Massachusetts USA) and spun in a microcentrifuge at 13,000 rpm for 15 min. The resulting supernatant was used to assay luciferase activity and total protein.

### Protein quantitation

Total protein was assayed in cell lysates after Bradford with modifications [27, 28] using 96-well plates (Corning, Corning, New York, USA) and an Infinite M200 PRO plate spectrophotometer (Tecan, Männedorf, Zürich, Switzerland). The staining solution contained 0.03% Coomassie G-250 (LOBA Feinchemie, Fischamend, Austria), 5% of ethanol, and 10% of phosphoric acid (Chimmed, Moscow, Russia). The calibration curve was plotted using bovine IgG (Reanal, Budapest, Hungary) as the standard.

### Luciferase activity assay

Luciferase activity was determined in cell lysates following the recommendation of the Luciferase Assay System manufacturer (Promega, Madison, WI, USA) using 96-well luminescence plates (Corning Incorporated, Corning, New York, USA) and an Infinite M200 PRO plate spectrophotometer (Tecan, Männedorf, Zürich, Switzerland). The substrate solution contained 1 mM of D-luciferin of the firefly *P*. *pyralis* (Promega, Madison, WI, USA), 25 mM of Tris-phosphate, 50 mM of 2-mercaptoethanol, 2.5 mM of EDTA, 10 mM of MgSO_4_, (Amresco, Radnor, PA USA), and 4 mM of dATP (AppliChem, Darmstadt, Germany), pH 7.8.

### *D*. *rerio* maintenance

Zebrafish were kept in a flow-through aquarium (Aqua Schwarz, Göttingen, Germany) at 28°C. Conforming to the international standards, the light/dark cycle was 14 h light / 10 h dark. Adult fish were fed once a day with brine shrimps *Artemia salina* (Barrom, Barnaul, Russia) and dry feed Sera Vipan (Sera, Immenhausen, Germany).

Fish fry of 4 days and more after hatching were fed twice a day with a suspension of the paramecia *Paramecium caudatum* or dry feed Sera Micron (Sera, Immenhausen, Germany). A line of wild-type AB *D*. *rerio* was used.

The experiments on animals were carried out in strict accordance with the regulations of the European Convention for the Protection of Vertebrate Animals used for Experimental and other Scientific Purposes (ETS No. 123) and bioethical principles (https://cioms.ch/images/stories/CIOMS/IGP2012.pdf). All experimental procedures were performed in accordance with the World Animal Protection standards and were approved by the Research Ethics Committee (#12–2017) of the Institute of Molecular Genetics, Russian Academy of Sciences.

### DNA microinjection into the developing *D*. *rerio* embryo

The samples were injected into the *D*. *rerio* embryos at the first cleavage division 30 min after fertilization using a micromanipulator (model M-152, Narishige Scientific Instrument Lab, Tokyo, Japan) and a Pneumatic PicoPump (PV820, World Precision Instruments, Sarasota, FL, USA) under an inverted microscope Olympus IX2-SLP (Olympus, Tokyo, Japan).

The capillaries with the outer diameter of 20 μm were obtained from the capillaries (cat.# BF100-50-10, Sutter Instrument Company, Novato, CA, USA) on a micropipette puller (Sutter Instrument Company, Novato, CA, USA). The embryo was injected with 1 nl of a sample containing from 1 to 100 pg of DNA per embryo. DNA was diluted in PBS (Biolot, Moscow, Russia) containing 0.05% phenol red (Sigma-Aldrich, St. Louis, MO, USA) in the final mixture.

At least three independent experiments were carried out for each analyzed genetic construct and DNA dose injected into *D*. *rerio* eggs. The number of eggs injected with each DNA dose varied from 100 to 120.

### Detection of EGFP-positive cells in the developing *D*. *rerio* embryo

The time-related detection of EGFP-positive cells in the developing embryo was analyzed for 10 days using an upright fluorescence microscope Leica DM 1000 and a ICC50 HD camera (Leica Microsystems, Wetzlar, Germany) with a fluorescence module (Leica GFP ET cube, Ex 470/40 nm, Em 525/50 nm) at magnifications from ×50 to ×100.

The embryos were preliminarily anesthetized with 0.006% water solution of tricaine (Sigma-Aldrich, St. Louis, MO, USA).

**Statistical processing** of experimental data was performed using MS Excel 2007 (Microsoft Corporation, Redmond, WA, USA) and RStudio version 3.3.3 (RStudio, Inc.). For repeated measurements, the one-way ANOVA with Tukey's correction was used comparison one-way ANOVA was used followed by Tukey post hoc method (α = 0.05).

## Results

### Luciferase activity provided by different vectors in HEK293 cells

**Fig 2:**
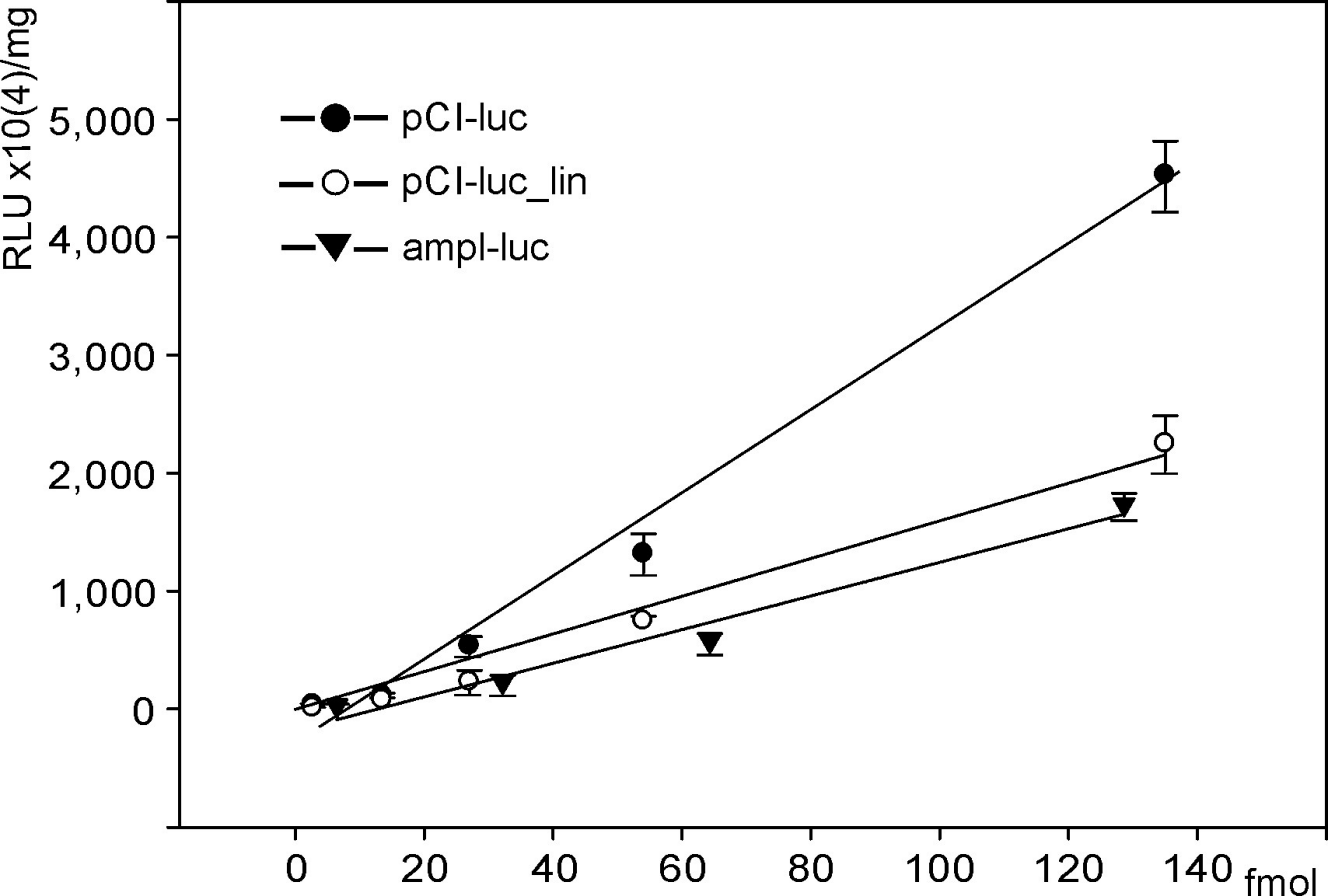
Luciferase activity in HEK293 cells transfected with pCI-luc, pCI-luc_lin, or ampl-luc. Abscissa: DNA quantities (femtomoles) of expression vectors pCI-luc, pCI-luc_lin, and ampl-luc used in transfection. Ordinate: luciferase activity normalized to protein quantity in lysates of HEK293 cells. The found relationships are described by linear equations y = (35.022x - 318)×10^4^ with the approximation reliability R^2^ = 0.9887 for pCI-luc; y = (17.514x - 148.92)×10^4^ with R^2^ = 0.9928 for pCI-luc_lin; and y = (14.252x - 181.65)×10^4^ with R^2^ = 0.9723 for ampl-luc. Luciferase activity provided by the constructed vectors was compared using the one-way ANOVA with Tukey's correction for multiple comparisons. Significant differences in luciferase activity were observed for the pCI-luc vs pCI-luc_lin and the pCI-luc vs ampl-luc groups when the vector quantity exceeded 27 fmol. RLU, relative luminescence unit.

The circular plasmid pCI-luc, its linear form pCI-luc_lin, and the vector carrying a similar expression cassette with the amplified DNA fragment ampl-luc were used in the experiments (Fig 1). In order to provide uniform transfection conditions [26] for different quantities of introduced expression constructs, an approach maintaining a constant amount of DNA in the transfection mixture based on combining the target vector and ballast DNA was used, which allowed us to widely vary the number of target constructs. Analysis of changes in luciferase activity as a function of molar concentrations of the genetic constructs (Fig 2) demonstrates close-to-linear relationships for all analyzed vectors. In the range of DNA concentrations used, the average efficiency of the circular vector was 2.5 times those of the linear and PCR- amplified fragment.

The effect of mutations on the efficiency of the PCR- amplified fragment was evaluated by cell transfection by the same quantities of the vector obtained using a different number (from 10 to 35) of polymerase chain reaction cycles.

**Fig 3:**
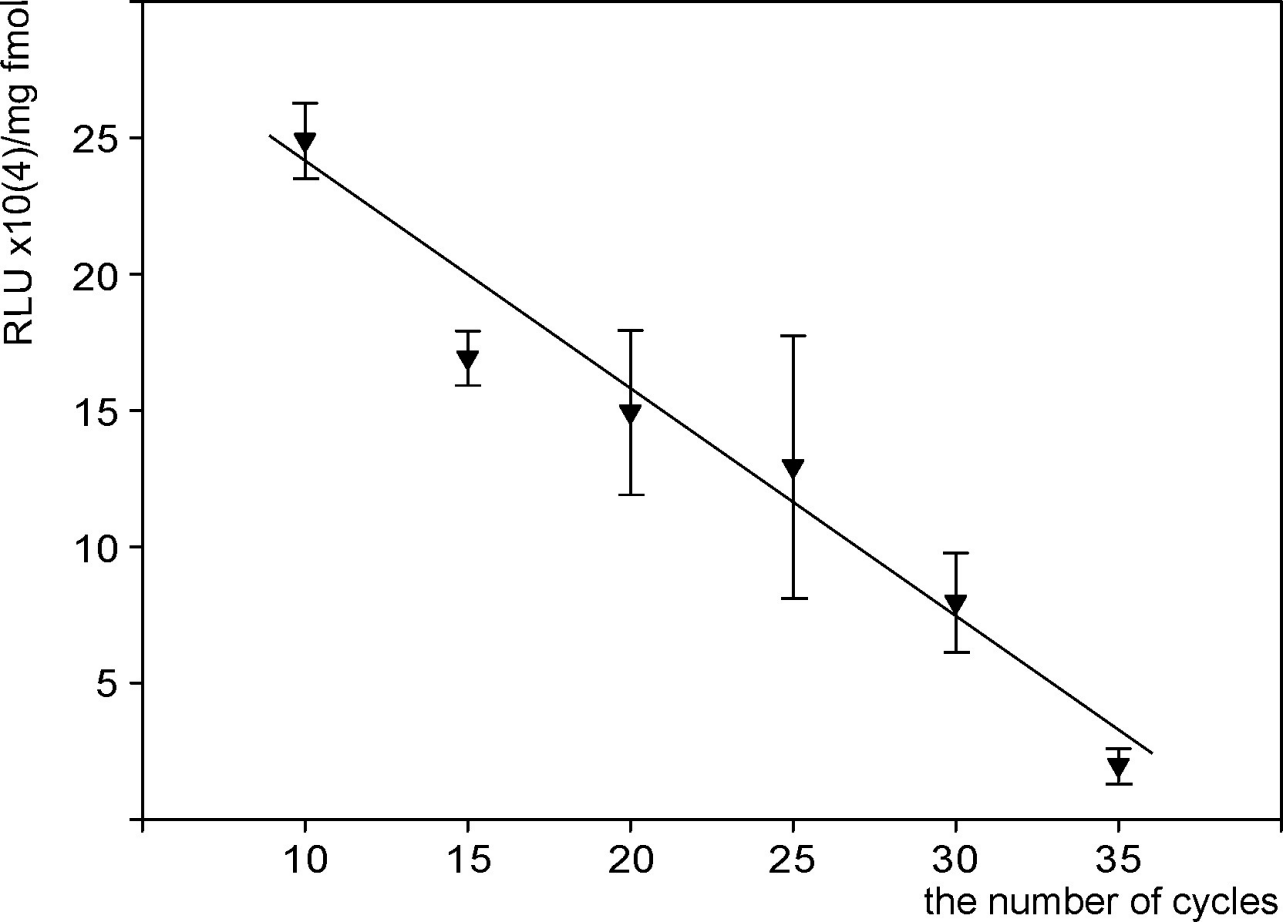
Accumulation of luciferase activity in HEK 293 cells after transfection by ampl-luc variants obtained using different number of PCR cycles. Abscissa: number of PCR cycles used to synthesize ampl-luc. Ordinate: luciferase activity detected in lysates of HEK293 cells 48 h after transfection. The activities were normalized to the quantities of protein in lysates and vector DNA (femtomoles) used in transfection. Transfection was carried out using 500 ng (321.5 femtomoles) of ampl-luc DNA per 50,000 HEK293 cells. The found relationship is described by the equation y = (-0.8203x + 31.75)×10^4^ with the approximation reliability R^2^ = 0.9576.

Luciferase activity decreased in cells almost linearly with the number of PCR-cycles (Fig 3), and can be approximated by the function y = (-0.8203x + 31.75)×10^4^ with R^2^ = 0.9576.

Additional data on the effect of mutations on the expression efficiency of the PCR- amplified fragment were obtained by cloning individual DNA fragments after 25 PCR cycles into рUC19. Such constructs were used to transfect HEK293 cells to assay their luciferase activity (Table 1).

**Table 1:** Description of constructs obtained by cloning of individual PCR fragments into pUC19. Positions of mutant or deleted nucleotides in the promoter/operator, luciferase gene, and polyadenylation site are numbered from the beginning of the corresponding region. The standard deviation is indicated in parentheses.

Clone number	Luciferase activity, RLU×10^4^/(mg×fmol)	Identified mutations
1	0.041 (± 0.02)	Substitutions in the *luc*, G319/A and Asp107/Asn
2	0.00022 (± 3.77E-05)	A466 deletion in the coding region of *luc* leading to a frameshift and a stop codon 114 bp downstream of
3	0.018 (± 0.065)	Mutation in the *luc* with synonymous substitutions in T132/A and T561/G.
		T insertion after position 696 in the coding region of *luc* leading to a frameshift and a stop codon 101 bp downstream of it
4	0.478 (± 0.228)	Point substitution in the promoter G1217/A
5	0.009 (± 0.004)	An A insertion after position 534 leading to a stop codon 26 bp downstream of it
6	28.45 (± 3.668)	Point substitution in the promoter T612/C. Mutation in the *luc* with synonymous substitutions in C1149/T and C1581/A
7	12.724 (± 0.905)	Point mutation C125/T in the polyadenylation site
8	1.573 (± 0.383)	Point substitution in the promoter T95/C. Mutation in the *luc* with synonymous substitutions in C1149/T and C1581/A
9	2.006 (± 0.341**)**	Point mutation T713/A in the coding region of *luc* leading to a Leu238 to stop substitution
10	4.136 (± 0.231**)**	Point substitution in the enhancer A196/G. Point substitution in the promoter G247/A
pCI-luc	33.392 (± 2.118)	No mutations
pUC19	6.972E-05 (± 1.57E-05)	

The results obtained significantly varied between the tested constructs. For instance, p6 and p7 provided for luciferase activity similar to that of the initial pCI-luc. No activity was detected for p2, p3, and p5; while it was 800 times lower for p1 vs pCI-luc; 70 times lower, for p4; and 8–20 times lower, for p8, p9, and p10. All these cloned PCR fragments were sequenced, and point mutations were found in their sequences. The Asp107/Asn mutation underlies the radical decrease in the enzyme activity in p1. Frameshift deletions in the coding region of *luc* are responsible for the complete luciferase inactivation in cells transfected with p2, p3, and p5. In some cases, the altered expression efficiency was due to mutations in the regulatory regions of the gene. Thus, the promoter region of p4 had the G1217/A substitution providing for a considerable decrease in luciferase activity relative to pCI-luc. At the same time, the T612/C mutation in the promoter region coupled with synonymous substitutions C1149/T and C1581/A in the coding region had no notable effect on the expression efficiency of p6. The C125/T mutation in a polyadenylation site of p7 decreased the enzyme activity roughly thrice. A 20-fold decrease in the efficiency of p8 was due to the T95/C substitution in the promoter region, which was accompanied by synonymous C1149/T and C1581/A substitutions in the coding region as in p6. Point substitutions A196/G and G247/A in the enhancer and promoter regions of p10, respectively, decreased luciferase activity 8-fold. The case of p9 carrying a stop codon substitution (TTA/TAA or Leu238/stop) in the coding region remains enigmatic. Despite this mutation, luciferase activity is clearly detected in transfected cells at the level of ~6% relative to pCI-luc. Presumably, HEK293 cells can to some extent suppress this translation termination signal.

### Generation of EGFP-positive cells in developing embryos

**Fig 4:**
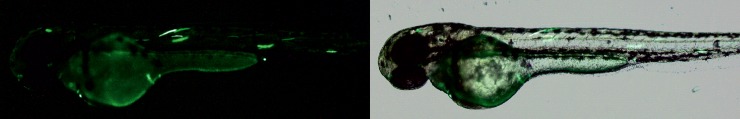
Detection of EGFP-positive cells in developing *D*. *rerio* embryos. Fluorescent (left) and bright/fluorescent fields (right) images are presented. 50 × magnification. The embryos at 2 days post-fertilization were injected with pCI-EGFP (0.013 fmol).

EGFP-positive cells could be detected one day after the injection of pCI-EGFP and ampl-EGFP into the yolk of *D*. *rerio*. Their number peaked 2 days after the injection for all DNA doses used and gradually decreased later (Fig 4).

**Fig 5:**
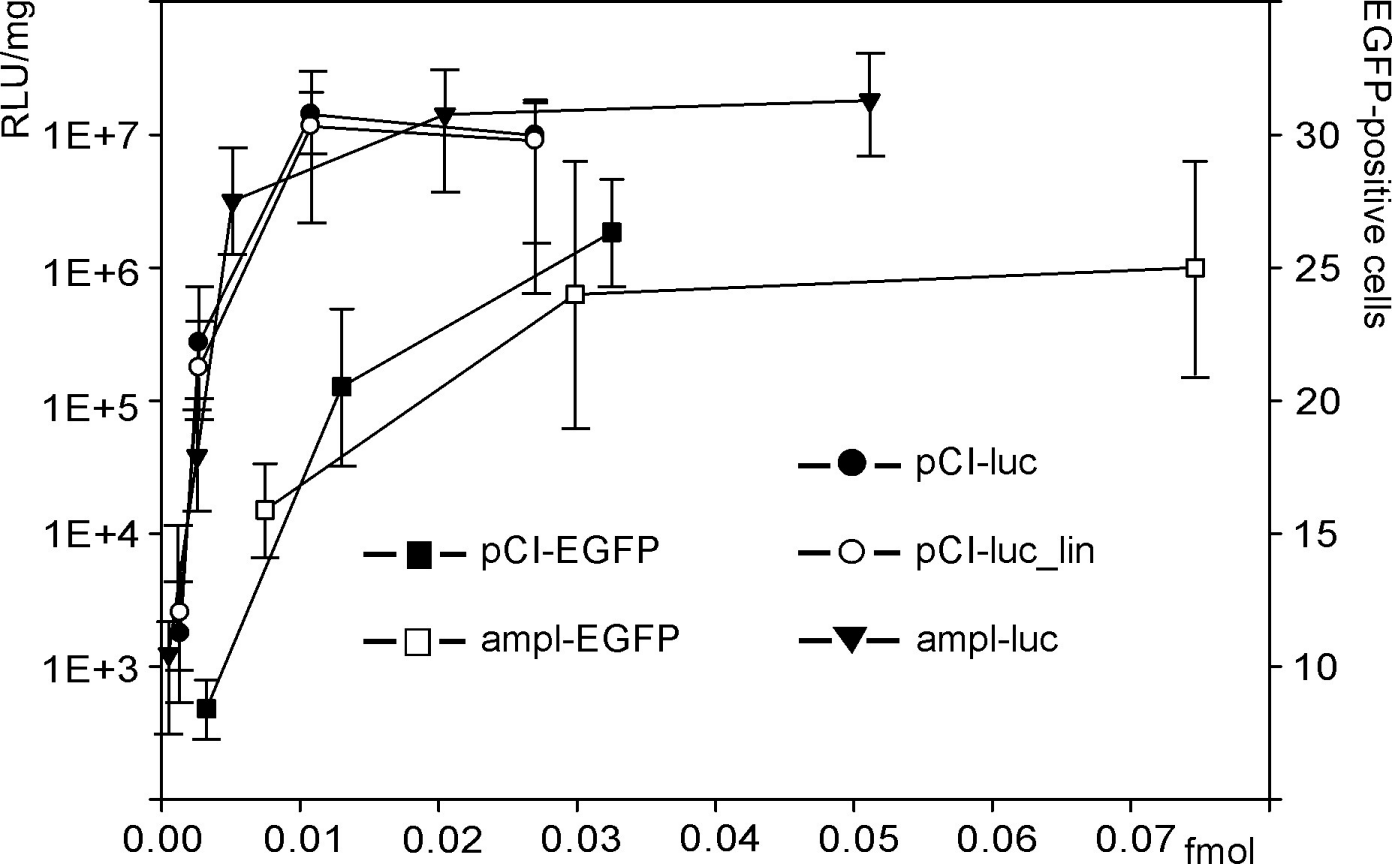
EGFP-positive cells and luciferase activity in cells lysates of developing *D*. *rerio* embryos. Abscissa: injected DNA quantity (femtomoles) of expression vectors pCI-EGFP, ampl-EGFP, pCI-luc, pCI-luc_lin, and ampl-luc. Ordinate, left: luciferase activity (logarithmic scale) normalized to total protein detected in lysates of *D*. *rerio* embryos 72 h after injection. Ordinate, right: the number of EGFP-positive cells per embryo 48 h after injection. The one-way ANOVA with Tukey's correction for multiple comparisons was used to compare luciferase activities provided by the constructed vectors as well as the number of EGFP-positive cells in developing *D*. *rerio* embryos. No significant differences in luciferase activity have been found in *D*. *rerio* cell lysates after the transfection with pCI-luc, pCI-luc_lin, and ampl-luc as well as in the number of EGFP-positive cells after the transfection with pCI-EGFP and ampl-EGFP.

The relationship between the number of EGFP-positive cells in the embryos and the molar quantities of the expression constructs (Fig 5) does not significantly differ for the plasmid and the PCR- amplified fragment.

### Luciferase activity in cell lysates of developing *D*. *rerio* embryos

Luciferase activity was observed 24 h after the injection of pCI-luc, pCI-luc_lin, and ampl-luc into the yolk of *D*. *rerio* and peaked at 3–4 days. Both for plasmid constructs and PCR- amplified fragment, a noticeable increase in luciferase activity was observed after their injection in the range from 5×10^−4^ to 10^−2^ femtomoles (3×10^5^ to 6.5×10^6^ molecules), after which they reach the plateau at the same level. No significant difference in the expression efficiency of the analyzed constructs has been revealed.

## Discussion

To compare the expression efficiency of plasmid vector and PCR- amplified construct *in vitro* the circular and linear form of plasmid as well as the vector carrying a similar expression cassette inside the amplified DNA fragment were introduced into HEK293 cells. In the case of *in vitro* transient transfection, the quantity of the introduced vector molecules can be of great significance. Excessive amounts of the expression constructs can oversaturate the transcription and/or translation systems [29, 30]. In this event, the product concentration reflects the expression capacity of the cell rather than the efficiency of the genetic construct.

The near-linear relationship between the molar concentration of the introduced genetic material and the reporter protein accumulation observed in the used system (Fig 2) indicates that the protein synthesis system or the systems of vector transport into the cytoplasm and nucleus are not exhausted. Thus, the difference in the luciferase activity in cells likely reflects the different efficiency of the used genetic constructs.

The findings suggest that the efficiency of the circular plasmid was roughly 2.5 times higher than those of the linear plasmid and the PCR- amplified fragment. PCR- induced mutations in the expression cassette could be an obvious reason for the decline of the PCR- amplified fragment efficiency relative to the circular plasmid. These data coincide with our previous data on the expression efficiencies of the plasmid and a PCR construct based on the *EGFP* gene [24]. In this work, HEK293 cells were transfected with different quantities of tested constructs using the ballast DNA method, and EGFP accumulation was evaluated by flow cytofluorometry. This demonstrated a ~threefold transgene expression level compared to the PCR vector, which corresponds to the results reported here.

However, it should be noted that the efficiency of the linear plasmid and the PCR- amplified fragment are quite similar. It is unexpected considering the presence of mutations in the latter vector. This fact would seem to indicate that the mutations are insignificant for the active protein accumulation provided by the PCR- amplified fragment and points to the involvement of other factors, such as DNA unwinding or sensitivity of linear DNA to nucleases [17].

The influence of acquired mutations on the decrease in the expression efficiency was evaluated quantitatively by cell transfection using the vectors obtained after different numbers of PCR cycles (Fig 3). The relationship between the luciferase activity (y) and the number of PCR cycles (x) can be approximated by the equation y = (-0.8203x + 31.75)×10^4^, R^2^ = 0.9576.

If this equation is fulfilled beyond the specified range, its extrapolation to х = 0 gives us a the conditional specific value of luciferase activity accumulation (roughly 31.75×10^4^ RLU/(mg×fmol)), that would be observed in case of complete absence of mutations in the PCR product. Taking this value as 100%, we can evaluate the decrease in the activity of the marker enzyme associated with the accumulation of mutations in the expression cassettes after different numbers of PCR cycles.

The mutations generated after 25 cycles should decrease the cellular luciferase activity by ~65%. This estimate coincides with the 70% decrease in the luciferase activity provided by the PCR- amplified vector relative to the circular plasmid (Fig 2).

Similar data on the impact of mutations on the activity of PCR- amplified fragment were obtained in experiments evaluating the expression efficiency of individual DNA fragments obtained by PCR and cloned into pUC19.

Averaged for 10 analyzed variants, the level of luciferase activity observed upon transfection of HEK 293 cells by these constructs (Table 1) was 4.9±0.6×10^4^ RLU/(mg×fmol), which is about 15% of that for pCI-luc. This value is also a good fit to the above data on the decreased target activity provided by the PCR- amplified fragment relative to the circular plasmid.

Overall, the obtained data indicate that the accumulation of mutations in the expression cassette is the key factor causing the decreased expression efficiency of the PCR- amplified fragment relative to the circular plasmid in *in vitro* experiments with a polycationic transfection reagent, while other factors have no comparable effect.

At the same time, similar marker gene activities provided by the linearized plasmid and PCR- amplified construct are intriguing. The mechanisms causing the decreased efficiency of the linear plasmid relative to the circular one without having a noticeable effect on the functioning of the linear PCR vector remain unclear. We can speculate that it is due to less efficient nuclear import of the linear plasmid relative to its more compact circular form and a shorter PCR- amplified fragment.

The dynamics of EGFP-positive cells accumulation in the *D*. *rerio* embryos coincided for the plasmid and PCR- amplified fragment (Fig 5). In both cases, the number of EGFP-positive cells simultaneously increased for the vector range from 2×10^6^ to 2×10^7^ molecules per embryo, after which they reached the plateau at the same level. The number of embryonic cells with the fluorescence level detectable under experimental conditions is relatively low, on average 20–30 per embryo (Fig 4). However, the total number of cells expressing the fluorescent marker may not be limited to the observed EGFP-positive cells. The fluorescence intensity of individual cells can substantially vary even after microinjection of the same doses of genetic constructs into the nucleus [31].

A similar pattern was observed after transient transfection of HEK293 cells: the fluorescence intensity varied by several orders of magnitude in individual cells [24]. This pattern can be applicable to the functioning of the expression vector *in vivo*. It is conceivable that a substantial number of cells not detected as EGFP-positive under experimental conditions still contribute to the total level of the marker protein in the animal body. In this context, the assay for the total transgene expression product in the embryo can be more informative to compare the vector constructs.

Time-related changes of the enzyme activity in embryonic cell lysates after the injection of genetic constructs carrying the luciferase gene largely corresponded to the data obtained for the EGFP-expressing constructs.

At the same time, the plateau was reached at a smaller number of molecules for the constructs carrying the luciferase gene compared to those with the *EGFP* gene (Fig 4). For instance, the injection of 6.5×10^6^ to 2×10^7^ molecules per embryo for pCI-EGFP and ampl-EGFP notably increased the number of EGFP-positive cells. It likely indicates an increasing DNA uptake by the cells. At the same time, the injection of pCI-luc, pCI-luc_lin, and ampl-luc in the same dose range did not increase luciferase activity in the embryonic lysates.

Since the results obtained for constructs based on plasmids and PCR products carrying the same genetic markers coincide, the differences between the constructs providing for the expression of EGFP and luciferase are likely due to the nature of the marker and the assay system, here, the estimation of the number of EGFP-positive cells and marker enzyme activity. Thus, the data on the doses of genetic constructs providing for the maximum target production may differ when using different experimental approaches to characterize the corresponding expression constructs.

Herewith, no statistically significant difference in the expression efficiency of constructs based on plasmids and PCR vectors (Fig 5) was found at the organism level in the case of the *luc* marker gene, as well as *EGFP*.

Notice that the experimental data scatter is expectedly higher in the *in vivo* system relative to the simpler *in vitro* one. It does not allow us to reliably identify detailed differences in the efficiency of the vectors at the organism level. Apparently, certain properties of individual embryos’ response to the injection of analyzed genetic constructs are more significant in this system than the relatively minor differences in their expression efficiency.

## Conclusions

The results indicate that the constructs based on PCR- amplified DNA fragments are practically equal to the traditional plasmids in the expression efficiency. Productivity of the circular plasmid vector estimated on the *in vitro* model is just 2.5 times higher on average than that of the PCR- amplified fragment, synthesized by *Taq* DNA polymerase.

The key factor of the decreased expression efficiency of the PCR- amplified fragment was the accumulation of mutations in the expression cassette during the PCR reaction. This factor can be substantially decreased by using more accurate polymerases.

No significant difference in the expression efficiency of the analyzed constructs has been revealed in the *in vivo* model.

At the same time, the comparison of the efficiency observed for pCI-luc, pCI-luc_lin, and ampl-luc in the *in vivo* and *in vitro* systems in the dose ranges demonstrating noticeable luciferase activity dependence on the DNA quantity (Figs 2 and 5) indicates that in *D*. *rerio* cell lysates the accumulation value of luciferase activity, normalized by the molar amount of the used genetic constructs, was approximately three orders of magnitude higher than that after the transfection of HEK293 cells *in vitro*.

Apparently, this could be associated with the low efficiency of the interaction between the transfected complexes and cells in the *in vitro* system [24]. However, the results indicate additionally that the *in vitro* quantitative results cannot be extrapolated straightforwardly to the organism level.
